# Determination of Varying Group Sizes for Pooling Procedure

**DOI:** 10.1155/2019/4381084

**Published:** 2019-04-01

**Authors:** Wenjun Xiong, Hongyu Lu, Juan Ding

**Affiliations:** School of Mathematics and Statistics, Guangxi Normal University, Yucai Road 15, Guilin 541004, China

## Abstract

Pooling is an attractive strategy in screening infected specimens, especially for rare diseases. An essential step of performing the pooled test is to determine the group size. Sometimes, equal group size is not appropriate due to population heterogeneity. In this case, varying group sizes are preferred and could be determined while individual information is available. In this study, we propose a sequential procedure to determine varying group sizes through fully utilizing available information. This procedure is data driven. Simulations show that it has good performance in estimating parameters.

## 1. Introduction

Routine monitoring or large scale of screening usually occurs in biomedical research to identify infected specimens [1–4]. However, some test kits, e.g., nucleic acid amplification test (NAAT), are expensive [2, 5]. Therefore, the expense during a large-scale monitoring process is usually a financial burden if resource is limited [6–8]. The strategy of pooling biospecimens is attractive to address this issue [9–11], which was first used during World War II to screen for syphilis [12]. This strategy is firstly to pool specimens into groups and then screen these groups. If a group tests negative, all specimens in this group will be declared negative; otherwise, continue to perform individual test. When the prevalence is low, the total number of tests using pooling will be far less than that using the individual test. Due to its efficiency and cost saving, pooling is now applied in many fields, such as agriculture [13], genetics [14, 15], HIV/AIDS [16, 17] and blood screening [18], and environmental epidemiology [19, 20].

The gain of pooling mainly depends on the pooling algorithm. Assuming homogeneity of the population, dozens of papers have investigated the problem how to design an efficient algorithm [21–25]. However, this assumption might be violated in practical application [26–28]. While individual information is available, it is of interest to estimate individual-level prevalence through incorporating such information. Note that only group-level status is observed, e.g., positive or negative. This problem has been studied in parametric context through the framework of binary regression models [29–31], and also in semiparametric [32, 33] or nonparametric context [34, 35]. However, aforementioned work mostly uses a single group size that is determined in advance.

A set of pool sizes might be more appropriate while considering population heterogeneity. For example, varying pool sizes were used to estimate the infection prevalence of *Myxobolus cerebralis*, which causes whirling disease, among free-ranging salmonid fish collected from the Truckee River in Nevada and California [36]. In a study of estimating the prevalence of several viruses in carnations grown in nursery glasshouses in Victoria, sequential pooled testing involving several pool sizes was adopted [37]. Using a single group size might be optimal for some estimates but far from others, especially when we have little information ahead of the experiment [37, 38]. More work is better on this issue since the benefit of pooling algorithm mainly depend on the choice of pool size [38–40]. In this study, we propose a pooling strategy with varying pool sizes through taking advantage of individual information. Our procedure is a data-driven pooling algorithm, where groups are formed sequentially. Its performance is extensively investigated by simulations and a real data set.

## 2. Methods

### 2.1. Notations and Background

Suppose *N* specimens are assigned into *m* groups each with size *k*_*i*_ for *i*=1,2,…, *m*. *z*_*i*_ denotes the observed status of the *i* ^th^ group, and *X*_*ij*_ denotes the covariates of the *j* ^th^ specimen in the *i* ^th^ group for *j*=1,…, *k*_*i*_ and *i*=1,…, *m*. The observations are {*z*_*i*_, *X*_*ij*_, *j*=1,…, *k*_*i*_, *i*=1,…, *m*}, where *X*_*ij*_={1, *x*_1,*ij*_,…,*x*_*d*−1,*ij*_}^T^. Here, the notation *A* ^T^ represents the transpose of matrix *A*. The sensitivity and specificity of the screening tool are denoted by *S*_e_ and *S*_p_, respectively. The full likelihood function is

(1) $$\begin{array}{c} L\left(\beta ;z,X\right)=\overset{m}{\underset{i=1}{\prod }}{\left[S_{\text{e}}-r\overset{k_{i}}{\underset{j=1}{\prod }}\left(1-p_{ij}\right)\right]}^{z_{i}}{\left[1-S_{\text{e}}+r\overset{k_{i}}{\underset{j=1}{\prod }}\left(1-p_{ij}\right)\right]}^{1-z_{i}}, \end{array}$$

where *r*=*S*_e_+*S*_p_ − 1 and *p*_*ij*_=*g*(*β*_0_+*β*_1_ *x*_1,*ij*_+⋯+*β*_*d*−1_ *x*_*d*−1_, *ij*)=*g*(*X*_*ij*_^T^ *β*). The parameter *β* is defined by *β*={*β*_0_, *β*_1_,…,*β*_*d*−1_}^T^, and the function *g* ^−1^(·) is a known, monotone, and differentiable link function.

Sometimes there might be a maximum admissible group size *k* ^max^, e.g., a large group size might bring the dilution effect. Therefore, we should carefully choose an appropriate group size that is smaller than *k* ^max^. Define a set *𝒦*={1,2,…, *k* ^max^}, and denote it by **k**={*k*_1_,…, *k*_*m*_}, *k*_*i*_ ∈ *𝒦*, *i*=1,…, *m*. Once the group size **k** is determined, we could obtain the estimator of *β* through maximum likelihood function *L*(*β*, *z*, *X*). The Fisher information matrix of the parameter *β* could be rewritten as follows:

(2) $$\begin{array}{c} I\left(\beta ,k\right)=\overset{m}{\underset{i=1}{\sum }}\frac{G_{i}\left(k_{i},\beta \right)G_{i}^{\text{T}}\left(k_{i},\beta \right)}{C_{i}\left(\beta ,k_{i}\right)}, \end{array}$$

where

(3) $$\begin{array}{c} H_{i}\left(k_{i},\beta \right)=-\frac{1}{k_{i}}\overset{k_{i}}{\underset{j=1}{\sum }}\text{log }\left(1-g\left(X_{ij}^{\text{T}}\beta \right)\right),\\ G_{i}\left(k_{i},\beta \right)=\frac{\partial }{\partial \beta }H_{i}\left(k_{i},\beta \right),\\ C_{i}\left(\beta ,k_{i}\right)=\left(S_{\text{e}}-r{\text{exp}}^{-k_{i}H_{i}\left(k_{i},\beta \right)}\right)\left(1-S_{\text{e}}+r{\text{exp}}^{-k_{i}H_{i}\left(k_{i},\beta \right)}\right)r^{-2}k_{i}^{-2}{\text{exp}}^{2k_{i}H_{i}\left(k_{i},\beta \right)}. \end{array}$$

The calculation of Fisher information *I*(*β*, **k**) is presented in Supplemental Material (Available here). To obtain a better estimator $\hat{\beta }$, we try to find **k** that maximizes Fisher information *I*(*β*, **k**). However, individual-level measurements make it difficult to achieve this goal.

The Fisher information *I*(*β*, **k**) defined in (2) involves a measurement *H*_*i*_(*β*, *k*_*i*_), along with its functions *G*_*i*_(*k*_*i*_, *β*) and *C*_*i*_(*β*, *k*_*i*_). According to Delaigle and Hall [41], ∏_*j*=1_^*k*_*i*_^(1 − *g*(*X*_*ij*_^T^ *β*)) is generally close to ${\left(1-g\left({\bar{X}}_{i}^{\text{T}}\beta \right)\right)}^{k_{i}}$, where ${\bar{X}}_{i}=1/k_{i}\sum_{j=1}^{k_{i}}X_{ij}$. This closeness let the Fisher information reduce to the following format: *I*(*β*, **k**)=∑_*i*=1_^*m*^ *Z*_*i*_(*β*)*Z*_*i*_(*β*)^T^/*C*_*i*_(*β*, *k*_*i*_), where $Z_{i}\left(\beta \right)=g^{\prime }\left({\bar{X}}_{i}^{\text{T}}\beta \right){\bar{X}}_{i}/\left(1-g\left({\bar{X}}_{i}^{T}\beta \right)\right)$. Then, we propose to determine the group sizes through minimizing all *C*_*i*_(*β*, *k*_*i*_) with respect to *k*_*i*_ for *i*=1,…, *m*.

Note that the aforementioned approximate approach requires the pools are homogeneous. There are two methods to obtain homogeneous pool: reorder the specimens according to similarity of covariants or based on individual risk probability. The latter is adopted in this study. Following the method in McMahan et al. [42], the procedure of forming homogeneous pool is as follows. Firstly, use training data or prior knowledge to obtain an initial estimator *β* ^(0)^ [42]. Secondly, sort the specimens by their risk probability. Let *G* denotes the set which contains total covariants of enrolled specimens, *G*={**x**_1_,…, **x**_*N*_}, where *N* is the number of specimens and **x**_*i*_ is the covariant of the *i* ^th^ specimen. Sort *G* by risk probability *p*_*i*_=*g*(**x**_*i*_^T^ *β* ^(0)^) in the descending order, and obtain a sorted set *G* ^s^={**x**_1_^s^, ⋯, **x**_*N*_^s^}. The remaining procedure is directly performed on this sorted set.

### 2.2. Sequential Adaptive Pooling Algorithm

Our strategy is an adaptive design, which is often adopted in the biological experiment and also in the pooled test [22]. Before stating the algorithm, we need the following result. Suppose the specimens are assigned for the first *l* − 1 groups with the corresponding group sizes {*k*_1_,…, *k*_*l*−1_}. Let *n*_*l*_=∑_*j*=1_^*l*^ *k*_*j*_ for *l* ≥ 1 and *n*_0_=0. Denote *W*_*l*_(*β*)=−log(1 − *g*((**x**_*n*_*l*−1_+1_^s^)^T^ *β*)). Then the group size for the next group, *k*_*l*_, equals *k* ^max^ if *k* ^max^ ≤ *ϕ*_0_/*W*_*l*_(*β* ^(0)^). Here, *ϕ*_0_ is the root of an equation 2*S*_e_(1 − *S*_e_)(*ϕ* − 1)*e* ^2*ϕ*^+*r*(2*S*_e_ − 1)(*ϕ* − 2)*e* ^*ϕ*^+2*r* ^2^=0 and is approximately 1.8414. The proof of this result is presented in Supplemental Material (Available here). Our pooling strategy is described as follows:

*Step 1*. Label the specimens according to the ordering of *G* ^s^. For example, label the specimen with covariants **x**_1_^s^ by number 1. Assign specimens with labels up to *k* ^max^ into *l* ^th^ group.

*Step 2*. Calculate the corresponding function *C*_*l*_(*β* ^(0)^, *k*), *k* ∈ *𝒦* and *c*_0_=*ϕ*_0_/*W*_*l*_(*β* ^(0)^). If *k* ^max^ ≤ *c*_0_, defines *k*_*l*_ by *k* ^max^, choose the group size *k*_*l*_ which minimizes the function *C*_*l*_(*β* ^(0)^, *k*), *k*_*l*_=argmin_*k*∈*𝒦*_ *C*_*l*_(*β* ^(0)^, *k*). Define the set of covariants *G*_*l*_={**x**_*n*_*l*−1_+1_^s^,…, **x**_*n*_*l*__^s^}.

*Step 3*. Let *G* ^s^=*G* ^s^/*G*_*l*_, *l*=*l*+1. Repeat Step 2 to form the next group in the same way until all specimens are assigned.

*Step 4*. Screen the groups and obtain maximum likelihood estimator of *β*.

Note that this is a data-driven pooling strategy. Additionally, the above procedure does not strictly require that all specimens are enrolled before screening since the set *G* ^s^ is dynamic and could be renewed by new enrolled specimens.

### 2.3. Numerical Results

In this section, we proceed to evaluate the performance of our proposed procedure. Name it by PSV, which is pooling strategy with varied group sizes. For comparison, we also present the results of pooling strategy with a *s*ingle group size *k*, named by PSS(k). The group size *k* for PSS(k) is given in advance, e.g., *k*=5, 10, or could be determined by the average prevalence of those enrolled samples. For the latter, we determine the optimal single group size *k* ^*∗*^ by minimizing the variance of $\hat{p}$.

To investigate the performance of these methods, define the link function *g*(·) as the logistic function *g*(*u*)=1/(1+exp(−*u*)). Then, individual prevalence is obtained through the following model:

(4) $$\begin{array}{c} \text{log}\frac{p_{ij}}{1-p_{ij}}=\beta_{0}+\beta_{1}x_{1,ij}+\cdots +\beta_{d-1}x_{d-1,ij},\;i=1,\ldots ,m,j=1,\ldots ,k_{i}. \end{array}$$

**Figure 1:**
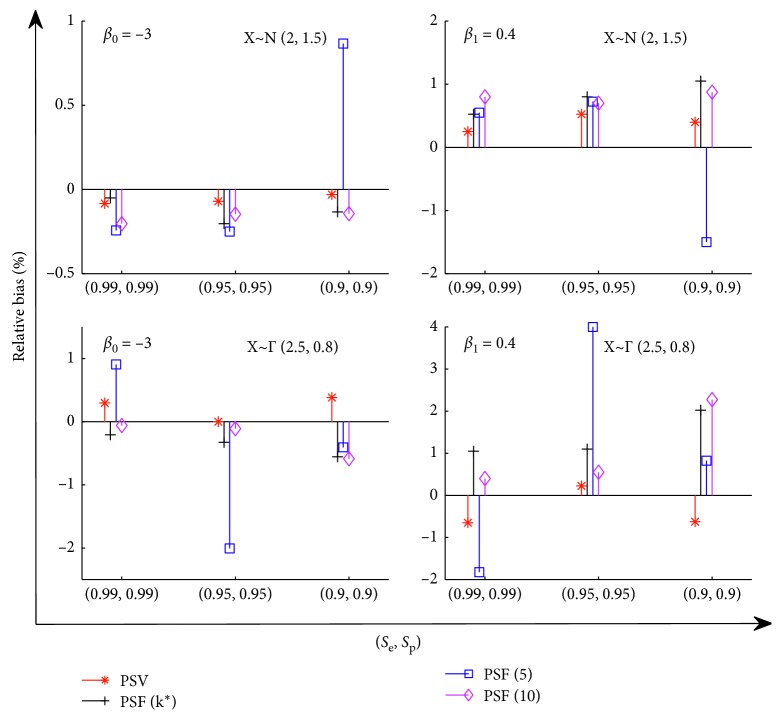
The relative bias of the parameters *β*_0_ and *β*_1_. The distribution of covariant is set by *N*(2,1.5) (top two panels) and Γ(2.5, 0.8) (bottom two panels), with the fixed number of groups *m*=1000.

We first consider a single covariant (*d*=2), following the normal distribution *N*(2,1.5) or the gamma distribution Γ(2.5, 0.8). The corresponding parameters are set by *β*_0_=−3 and *β*_1_=0.4. The samples are generated under these settings, and the procedures are repeated by *M*=5000 times. We report the estimators ${\hat{\beta }}_{0}$ and ${\hat{\beta }}_{1}$, along with their mean square error (MSE) in Table 1 under different settings of sensitivity, specificity, and the number of groups. In Figure 1, we further report the relative bias of the parameters.

Table 1 shows that all procedures have similar performance except PSF [5]. While using the procedure PSF, we have to choose a group size in advance. It is crucial for a group testing algorithm since the precision of estimators severely depend on the group size. In our setting, the average of individual prevalence is about 0.0997, and the corresponding optimal single group size is mostly *k* ^*∗*^=13, 12, 11 for (*S*_e_, *S*_p_)=(0.99, 0.99), (0.95, 0.95), and  (0.9, 0.9) respectively. Consequently, the procedure PSF [10] has better performance than PSF [5] since the latter procedure uses a too smaller group size. Figure 1 further shows the relative bias of the parameters, *β*_0_ and *β*_1_. Our procedure with varying group sizes, PSV, has very good performance under different scenarios. The procedure PSF [5] still has the poorest performance on the measurement of relative bias. As data-driven pooling strategies, PSV and PSF (*k* ^*∗*^) both show good performance, but PSV has smaller bias, which is a desired characteristic.

We proceed to consider the model (2) with *d*=4. Denote the single variable in the above setting by *x*_1_. We add two more variables: *x*_2_ follows the binomial distribution *B*(0.3) and *x*_3_ follows the normal distribution *N*(1,0.5). Then, the model (2) is

(5) $$\begin{array}{c} \text{logit}\left(p_{ij}\right)=\beta_{0}+\beta_{1}x_{1,ij}+\beta_{2}x_{2,ij}+\beta_{3}x_{3,ij},\;i=1,\ldots ,m,j=1,\ldots ,k_{i}. \end{array}$$

**Figure 2:**
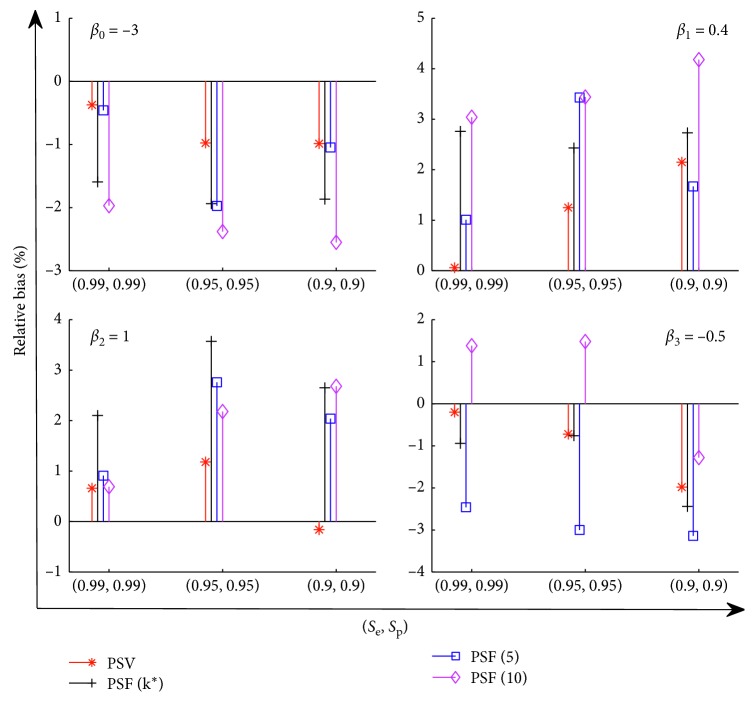
The relative bias of the parameters *β*_0_ − *β*_3_ under Model I: *x*_1_ ~ Γ(2.5, 0.8), *x*_2_ ~ *B*(0.3), and *x*_3_ ~ *N*(1,0.5), with the number of groups *m*=1000.

**Figure 3:**
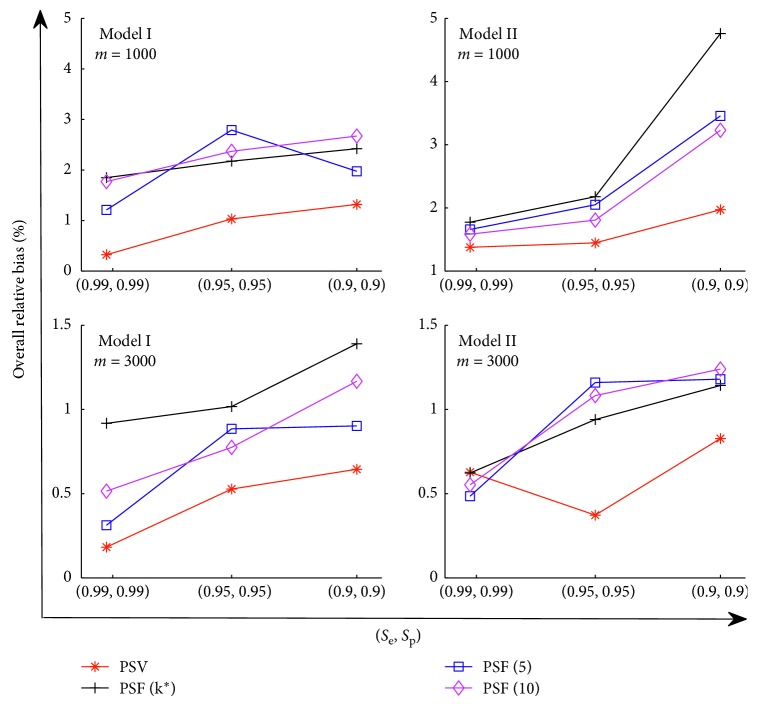
The overall relative bias of the parameters, defined as $R=\left(1/4\right)\sum_{l=1}^{4}\left|\left({\hat{\beta }}_{l}-\beta_{l}\right)/\beta_{l}\right|$. Model I: *x*_1_ ~ Γ(2.5, 0.8), *x*_2_ ~ *B*(0.3), and *x*_3_ ~ *N*(1,0.5). Model II: *x*_1_ ~ *N*(2,1.5), *x*_2_ ~ *B*(0.3), and *x*_3_ ~ *N*(1,0.5).

Specifically, denote by “Model I”: *x*_1_ ~ Γ(2.5, 0.8), *x*_2_ ~ *B*(0.3), *x*_3_ ~ *N*(1,0.5), and “Model II”: *x*_1_ ~ *N*(2,1.5), *x*_2_ ~ *B*(0.3), *x*_3_ ~ *N*(1,0.5). Set the parameters by *β*_0_=−3, *β*_1_=0.4, *β*_2_=1, and *β*_3_=−0.5. In Figure 2, we report the relative bias of the estimators ${\hat{\beta }}_{0}-{\hat{\beta }}_{3}$ under Model I. Furthermore, define a measurement of $R=\left(1/4\right)\sum_{l=1}^{4}\left|\left({\hat{\beta }}_{l}-\beta_{l}\right)/\beta_{l}\right|$ to calculate the overall relative bias. The results are reported in Figure 3.

Figure 2 shows that our procedure PSV performs best among the four procedures. It is a similar result as shown in Figure 1. The overall relative bias of these estimators reported in Figure 3 also confirms such property. It also reveals that pooling procedures using a single group size are not desired for a heterogeneous population, even the group size is carefully chosen, e.g., *k* ^*∗*^.

### 2.4. An Illustrative Application

Verstraeten et al. conducted a surveillance study in Kenya to monitor a trend in HIV risk over time [43]. The samples were collected from pregnant women, along with potential risk covariants such as age, parity, and education level. They used a common group size of 10 to estimate the seroprevalence of HIV. However, the individual prevalence of HIV is related with those risk covariants, e.g., the risk of HIV might tend to increase with age. For this data set, Vansteelandt et al. reported a set of group sizes varying between 5 and 12 under cost-precision trade-off [40].

We proceed to illustrate our pooling strategy based on part of these data published in [44]. They reported *N*=428 individuals enrolled in the experiment, including their age (*x*_1_) and education level (*x*_2_). Using model presented in [2], the individual prevalence *p*_*ij*_ follows the model: logit(*p*_*ij*_)=*β*_0_+*β*_1_ *x*_1,*ij*_+*β*_2_ *x*_2,*ij*_, *i*=1,…, *m*, *j*=1,…, *k*_*i*_ with *N*=∑_*i*=1_^*m*^ *k*_*i*_. Let the initial estimator be *β* ^(0)^=[−2, −0.05, 0.5]. Using our proposed pooling strategies PSV and PSF(*k* ^*∗*^), the group sizes are listed in Table 2. Correspondingly, we obtain estimators: $\hat{\beta }=\left[-2.909,-0.033,0.473\right]$ using PSV and $\hat{\beta }=\left[-3.011,-0.028,0.443\right]$ using PSF(*k* ^*∗*^).

## 3. Discussion

In biological and epidemiological studies, there is growing interest in developing methods for a more accurate result but less cost. Group testing is such a cost saving strategy. In this study, we developed a pooling strategy that uses varying group sizes while individual information is available. This strategy is attractive since it only depends on the information of enrolled specimens and does not require a group size chosen in advance. Due to the characteristic of data-driven and theoretical justification, the procedure, “PSV,” proposed in this study has a robust performance under different settings. It is convenient for practical application since we do not have to worry about how to choose an appropriate group size.

Varying group sizes are reasonable to be used when the target population is diverse. For example, a sequential testing procedure using several group sizes is adopted to estimate virus infection levels of carnation populations grown in glasshouses since different carnation populations were expected to have a wide range of infection levels [45]. We could pool more specimens into one group if the probability of testing positive is small. It sounds reasonable to balance the probability of testing positive for each group, a way to mimic the situation when all enrolled specimens are homogeneous.

In this study, we also propose a procedure using a single group size *k* ^*∗*^ determined by minimizing the variance of estimator of the prevalence. We could choose this procedure if we prefer a simple procedure or the diversity among the specimens to be screened is ignorable. Besides, we did not consider the cost of collecting specimens. If a test is much more expensive than that of collecting specimens, then the cost of tests is the main consideration in a project involving large-scale screening. Otherwise, it is necessary to take into account the overall cost of collecting and test while using the pooling strategy.

## Figures and Tables

**Table 1:** The performance of estimators using different pooling procedures.

(*S*_e_, *S*_p_)	*𝒜*	*m*=1000				*m*=500			
		*β*_0_		*β*_1_		*β*_0_		*β*_1_	
		Mean	MSE	Mean	MSE	Mean	MSE	Mean	MSE
*X*∼*N (2, 1.5)*									
(0.99, 0.99)	PSV	−3.003	0.020	0.401	0.002	−3.001	0.043	0.401	0.004
	PSF(*k* ^*∗*^)	−3.002	0.010	0.402	0.002	−3.006	0.022	0.403	0.004
	PSF(5)	−3.007	0.134	0.402	0.010	−3.018	0.289	0.405	0.021
	PSF(10)	−3.006	0.021	0.403	0.003	−3.009	0.042	0.403	0.005
(0.95, 0.95)	PSV	−3.002	0.026	0.402	0.003	−2.999	0.050	0.401	0.005
	PSF(*k* ^*∗*^)	−3.006	0.022	0.403	0.003	−3.009	0.041	0.406	0.006
	PSF(5)	−3.008	0.162	0.403	0.012	−2.997	0.317	0.400	0.023
	PSF(10)	−3.004	0.026	0.403	0.003	−2.998	0.052	0.401	0.006
(0.9, 0.9)	PSV	−3.001	0.034	0.402	0.003	−2.991	0.071	0.395	0.007
	PSF(*k* ^*∗*^)	−3.004	0.035	0.404	0.004	−3.007	0.074	0.404	0.010
	PSF(5)	−2.974	0.225	0.394	0.016	−2.993	0.418	0.399	0.031
	PSF(10)	−3.004	0.038	0.404	0.005	−3.008	0.077	0.404	0.010

*X* ∼ *Г (2.5, 0.8)*									
(0.99, 0.99)	PSV	−2.991	0.041	0.397	0.004	−2.997	0.020	0.399	0.002
	PSF(*k* ^*∗*^)	−3.006	0.020	0.404	0.003	−3.002	0.010	0.402	0.002
	PSF(5)	−2.973	0.281	0.393	0.020	−3.002	0.136	0.400	0.010
	PSF(10)	−3.002	0.042	0.402	0.005	−3.004	0.021	0.402	0.002
(0.95, 0.95)	PSV	−3.000	0.053	0.401	0.005	−2.998	0.026	0.400	0.003
	PSF(*k* ^*∗*^)	−3.010	0.041	0.404	0.006	−3.007	0.020	0.404	0.003
	PSF(5)	−3.060	0.324	0.416	0.023	−3.015	0.171	0.405	0.012
	PSF(10)	−3.003	0.053	0.402	0.007	−3.006	0.027	0.403	0.003
(0.9, 0.9)	PSV	−2.989	0.072	0.398	0.007	−2.992	0.034	0.399	0.004
	PSF(*k* ^*∗*^)	−3.017	0.075	0.408	0.010	−3.001	0.033	0.402	0.004
	PSF(5)	−3.012	0.379	0.403	0.028	−2.995	0.198	0.398	0.014
	PSF(10)	−3.018	0.075	0.409	0.010	−3.003	0.035	0.402	0.005

**Table 2:** The group sizes chosen using PSV procedure for the Kenyan example.

Procedure	PSV							PSS
Group size	6	7	8	9	10	11	12	11
Number of groups	2	2	2	4	3	4	23	39

## Data Availability

The Kenya data supporting this study are from previously reported studies and datasets, which have been cited. The data are available at https://cran.r-project.org/package=binGroup.

## Supplementary Materials

Supplementary Materials This article contains additional information on some technical aspects of the research, including the detailed calculation of the Fisher information matrix of the regression parameter and theoretical justification of Step 2 of our sequential adaptive pooling algorithm.
