# Supplementary material for: Determination of Varying Group Sizes for Pooling Procedure
Source: Comput Math Methods Med. 2019 Apr 1;2019:4381084. doi: 10.1155/2019/4381084 (PMC6466917; doi:10.1155/2019/4381084)
Supplement: Supplementary Materials — This article contains additional information on some technical aspects of the research, including the detailed calculation of the Fisher information matrix of the regression parameter and theoretical justification of Step 2 of our sequential adaptive pooling algorithm. [file 4381084.f1.pdf]

Online Supplementary Material for

On the Determination of Varying Group Sizes for Pooling Procedure

1 Calculation of the Fisher information matrix $I_{\beta,k}$

The log likelihood function is defined by $l = \log L$, where the likelihood function

$$L = \prod_{i=1}^{m} \left[S_e - r \prod_{j=1}^{k_i} (1 - g(X_{ij}^T \beta) \right]^{z_i} \left[1 - S_e + r \prod_{j=1}^{k_i} (1 - g(X_{ij}^T \beta) \right]^{1-z_i}.$$

We obtain the first and second derivative of l, which are

$$\frac{\partial l}{\partial \beta} = \sum_{i=1}^{m} \left[z_i \frac{r \sum_{l=1}^{k_i} g^{'}(X_{il}^T \beta) X_{il} \prod_{j \neq l} (1 - g(X_{ij}^T \beta))}{S_e - r \prod_{j=1}^{k_i} (1 - g(X_{ij}^T \beta))} - (1 - z_i) \frac{r \sum_{l=1}^{k_i} g^{'}(X_{il}^T \beta) X_{il} \prod_{j \neq l} (1 - g(X_{ij}^T \beta))}{1 - S_e + r \prod_{j=1}^{k_i} (1 - g(X_{ij}^T \beta))} \right],$$

and

$$\begin{aligned} \frac{\partial^2 l}{\partial \beta \partial \beta^T} =& \sum_{i=1}^{m} \left(\frac{z_i}{S_e - r \prod_{j=1}^{k_i} (1 - g(X_{ij}^T \beta))} - \frac{1 - z_i}{1 - S_e + r \prod_{j=1}^{k_i} (1 - g(X_{ij}^T \beta))} \right) \frac{\partial A_i(\beta, \mathbf{k})}{\partial \beta} \\ & - \sum_{i=1}^{m} \left(\frac{z_i}{(S_e - r \prod_{j=1}^{k_i} (1 - g(X_{ij}^T \beta)))^2} + \frac{1 - z_i}{(1 - S_e + r \prod_{j=1}^{k_i} (1 - g(X_{ij}^T \beta)))^2} \right) A_i(\beta, \mathbf{k}) A_i^T(\beta, \mathbf{k}), \end{aligned}$$

where $A_i(\beta, \mathbf{k}) = r \sum_{l=1}^{k_i} g^{'}(X_{il}^T \beta) X_{il} \prod_{j \neq l} (1 - g(X_{ij}^T \beta))$. Then the Fisher information matrix of the maximum likelihood estimator $\hat{\beta}$ is

$$\begin{aligned} I(\beta, \mathbf{k}) &= \sum_{i=1}^{m} \frac{A_i(\beta, k) A_i^T(\beta, k)}{(S_e - r \prod_{j=1}^{k_i} (1 - g(X_{ij}^T \beta)))(1 - S_e + r \prod_{j=1}^{k_i} (1 - g(X_{ij}^T \beta)))} \\ &= \sum_{i=1}^{m} \frac{(r \prod_{j=1}^{k_i} (1 - g(X_{ij}^T \beta)))^2}{(S_e - r \prod_{j=1}^{k_i} (1 - g(X_{ij}^T \beta)))(1 - S_e + r \prod_{j=1}^{k_i} (1 - g(X_{ij}^T \beta)))} \\ & \quad \cdot \sum_{l=1}^{k_i} \frac{g^{'}(X_{il}^T \beta)}{1 - g(X_{il}^T \beta)} X_{il} \sum_{l=1}^{k_i} \frac{g^{'}(X_{il}^T \beta)}{1 - g(X_{il}^T \beta)} X_{il}^T. \end{aligned}$$

Let $H_i(k_i, \beta) = -\frac{1}{k_i} \sum_{j=1}^{k_i} \log(1 - g(X_{ij}^T \beta)), \quad G_i(k_i, \beta) = \frac{\partial}{\partial \beta} H_i(k_i, \beta),$

$$C_i(\beta, k_i) = (S_e - r exp^{-k_i H_i(k_i, \beta)})(1 - S_e + r exp^{-k_i H_i(k_i, \beta)}) r^{-2} k_i^{-2} exp^{2 k_i H_i(k_i, \beta)}.$$

Then we have

$$I(\beta, \mathbf{k}) = \sum_{i=1}^{m} \frac{G_i(k_i, \beta) G_i^T(k_i, \beta)}{C_i(\beta, k_i)}.$$

□

2 Proof of Step 2

After determining the ith group size, we would like to choose the group size for the $i+1$ group. Let $W_{i+1}^s(k, \beta) = \frac{1}{k}\sum_{j=n_i+1}^{n_i+k} - \ln(1 - g(Z_j^{sT}\beta))$, then we have $\prod_{j=n_i+1}^{n_i+k}(1 - g(Z_j^{sT}\beta) = e^{-kW_{i+1}(k,\beta)}$ and

$$\begin{aligned}
C_{i+1}(\beta, k) &= \frac{(S_e - r\prod_{j=n_i+1}^{n_i+k}(1 - g(Z_j^{sT}\beta)))(1 - S_e + r\prod_{j=n_i+1}^{n_i+k}(1 - g(Z_j^{sT}\beta)))}{k^2 r^2 (\prod_{j=n_i+1}^{n_i+k}(1 - g(Z_j^{sT}\beta)))^2} \\
&= \frac{[S_e - re^{-kW_{i+1}(k,\beta)}][(1 - S_e) + re^{-kW_{i+1}(k,\beta)}]}{k^2 r^2 e^{-2kW_{i+1}(k,\beta)}} = \frac{[S_e e^{kW_{i+1}(k,\beta)} - r])[(1 - S_e)e^{kW_{i+1}(k,\beta)} + r]}{k^2 r^2} \\
&= k^{-2} r^{-2} \left[S_e(1 - S_e)(e^{k \cdot 2W_{i+1}(k,\beta)} - 1) + r(2S_e - 1)(e^{k \cdot W_{i+1}(k,\beta)} - 1) + S_p(1 - S_p) \right].
\end{aligned}$$

Denote $f(k, \delta) = k^{-2} r^{-2} \left[S_e(1 - S_e)(e^{k \cdot 2\delta} - 1) + r(2S_e - 1)(e^{k \cdot \delta} - 1) + S_p(1 - S_p) \right].$

The function $f(k, \delta)$ is a convex function of k. The proof is as follows.

$$\begin{aligned}
f(k, \delta) &= k^{-2} r^{-2} \left[S_e(1 - S_e)(e^{k \cdot 2\delta} - 1) + r(2S_e - 1)(e^{k \cdot \delta} - 1) + S_p(1 - S_p) \right] \\
&= \frac{S_e(1 - S_e)}{r^2} \sum_{j=1}^{+\infty} \frac{k^{j-2}(2\delta)^j}{j!} + \frac{2S_e - 1}{r} \sum_{j=1}^{+\infty} \frac{k^{j-2}\delta^j}{j!} + \frac{S_p(1 - S_p)}{k^2 r^2}.
\end{aligned}$$

Then the second derivative of the function $f(k, \delta)$ relative to k is

$$\begin{aligned}
\frac{\partial^2}{\partial k^2} f(k, \delta) = \frac{S_e(1 - S_e)}{r^2} \sum_{j=1}^{+\infty} \frac{(j-2)(j-3)k^{j-4}(2\delta)^j}{j!} \\
+ \frac{2S_e - 1}{r} \sum_{j=1}^{+\infty} \frac{(j-2)(j-3)k^{j-4}\delta^j}{j!} + 3! \frac{S_p(1 - S_p)}{k^4 r^2}.
\end{aligned}$$

Therefore, $\frac{\partial^2}{\partial k^2} f(k, \delta)$ is positive and thus the function $f(k, \delta)$ is a convex function of k.

Let ϕ_0 be the solution of the following equation $2S_e(1-S_e)(\phi-1)e^{2\phi}+r(2S_e-1)(\phi-2)e^{\phi}+2r^2=0$. Then we obtain $k_{opt}(\delta)=\text{argmin}_k f(k,\delta)=\phi_0/\delta$ for fixed δ. Then, we obtain that $f(k_1,\delta)\geq f(k_2,\delta)$ for $k_1\leq k_2\leq k_{opt}(\delta)$. Since $k_{opt}(\delta)=\phi_0/\delta$, then $k_{opt}(\delta_1)\geq k_{opt}(\delta_2)$ for $\delta_1\leq\delta_2$.

As described of the pooling strategy, $W_{i+1}^s(k,\beta)=\frac{1}{k}\sum_{j=n_i+1}^{n_i+k}-\ln(1-g(Z_j^{sT}\beta))$ with decreasing order series $g(Z_j^{sT}\beta), j=1,2,\cdots,N$. Thus, we have $W_{i+1}^s(k,\beta)\geq W_{i+1}^s(k_{\max},\beta)$ and $k_{opt}(W_{i+1}^s(k,\beta))\leq k_{opt}(W_{i+1}^s(k_{\max},\beta))$ for $k\leq k_{\max}$.

Let $W_{i+1}(\beta)=H_{i+1}^s(1,\beta)$ for simplicity and $k_{opt}^*=k_{opt}(W_{i+1}^s(\beta))$, $\delta_1=W_{i+1}(k,\beta)$ and $\delta_2=W_{i+1}(k_{\max},\beta)$, then $f(k,\delta_1)\geq f(k_{\max},\delta_1)$ for $k\leq k_{\max}\leq k_{opt}^*$. Since $f(k,\delta_1)\geq f(k,\delta_2)$ for $\delta_1\geq\delta_2$, we obtain that $f(k,\delta_1)\geq f(k_{\max},\delta_1)\geq f(k_{\max},\delta_2)$. Since $C_{i+1}(\beta,k)=f(k,H_{i+1}(k,\beta))$, we have that

$$C_{i+1}(\beta,k)\geq f(k_{\max},\delta_1)\geq C_{i+1}(\beta,k_{\max}),\text{for } k\leq k_{\max}\leq k_{opt}^*.$$

In this situation, we obtain that $k_{i+1}=\text{argmin}_{k\in\mathcal{K}}C_{i+1}(\beta,k)=k_{\max}$.

□
